# Analysis of variation at transcription factor binding sites in *Drosophila *and humans

**DOI:** 10.1186/gb-2012-13-9-r49

**Published:** 2012-09-05

**Authors:** Mikhail Spivakov, Junaid Akhtar, Pouya Kheradpour, Kathryn Beal, Charles Girardot, Gautier Koscielny, Javier Herrero, Manolis Kellis, Eileen EM Furlong, Ewan Birney

**Affiliations:** 1 European Bioinformatics Institute (EMBL-EBI), Wellcome Trust Genome Campus, Hinxton, Cambridgeshire, CB10 1SD, UK; 2 Genome Biol Unit, European Molecular Biology Laboratory, D-69117 Heidelberg, Germany; 3 MIT Computer Science and Artificial Intelligence Laboratory, Cambridge, MA 02139, USA; 4 Broad Institute, Cambridge, MA 02142, USA

## Abstract

**Background:**

Advances in sequencing technology have boosted population genomics and made it possible to map the positions of transcription factor binding sites (TFBSs) with high precision. Here we investigate TFBS variability by combining transcription factor binding maps generated by ENCODE, modENCODE, our previously published data and other sources with genomic variation data for human individuals and *Drosophila *isogenic lines.

**Results:**

We introduce a metric of TFBS variability that takes into account changes in motif match associated with mutation and makes it possible to investigate TFBS functional constraints instance-by-instance as well as in sets that share common biological properties. We also take advantage of the emerging per-individual transcription factor binding data to show evidence that TFBS mutations, particularly at evolutionarily conserved sites, can be efficiently buffered to ensure coherent levels of transcription factor binding.

**Conclusions:**

Our analyses provide insights into the relationship between individual and interspecies variation and show evidence for the functional buffering of TFBS mutations in both humans and flies. In a broad perspective, these results demonstrate the potential of combining functional genomics and population genetics approaches for understanding gene regulation.

## Background

Gene expression is tightly controlled by transcription factors (TFs) that are recruited to DNA *cis*-regulatory modules (CRMs). Many TFs have well-documented sequence preferences for their binding sites (transcription factor binding sites (TFBSs)) [1]. However, in contrast to the startling simplicity of the amino acid code, the 'regulatory code' at CRMs has a more ambiguous relationship between sequence and function. Chromatin immunoprecipitation (ChIP) coupled with genome-wide analyses have made it possible to map TF binding positions globally *in vivo*, which in some cases can serve as good predictors of CRM transcriptional outputs [2-4]. At the same time, these analyses often cannot explain the exact rules underlying TF binding to a given sequence, and functional prediction based on sequence alone has had limited success, in particular in mammalian systems [5].

Evolutionary analyses across species have proven to be a powerful approach in elucidating the functional constraints of DNA elements, in particular protein-coding genes, but are less interpretable in the context of CRM architecture [6,7]. In part, this is due to the fact that CRMs often have a 'modular', rather than 'base-by-base', conservation that may escape detection by conventional alignment-based approaches [8]. Moreover, conservation in DNA binding profiles can be detected even without apparent DNA sequence constraint [9]. Even at the level of individual TFBSs, differences in sequence may be hard to interpret - as such differences, for example, may reflect evolutionary 'fine-tuning' to species-specific factors to preserve uniform outputs rather than signifying a lack of functional constraint [6,10-12].

A complementary way to analyze the relationship between sequence and function is to explore intra-species (that is, polymorphic) variation of functional elements. Variation at DNA regulatory elements is relatively common and at least a fraction of it falls directly at TFBSs [13,14]. While some regulatory variants have been associated with major changes in transcription factor binding [15-17], gene expression [18,19] and disease phenotypes [20], many others do not result in apparent aberrations in function. This difference in itself suggests that analyzing TFBS variability in the context of the same species may lead to insights into *cis-*regulatory logic. For example, high tolerance of a binding site to deleterious variation may indicate that such variation is effectively 'buffered', either at the level of the same regulatory module or elsewhere in the system.

Until recently, large-scale population genomics studies of metazoan TFBSs were unthinkable because of the limited number of available genotypes and global TF binding profiles. However, advances in sequencing technology have paved the way for high-throughput efforts, such as the human 1000 Genomes project [21] and *Drosophila *Genetic Reference Panel (DGRP) [22], that are making available an increasing number of individual genomes originating from the same population. Combining these data with the binding maps of dozens of TFs in both species generated by the Encyclopedia of DNA Elements (ENCODE) for human [23], and modENCODE and other published sources in *Drosophila *[2,24-30] has provided an unprecedented resource for analyzing TFBS functional constraints.

Here we use three different approaches to take advantage of variation data in this context. First, we analyze TFBSs position-by-position to confirm that the levels of variation are generally consistent with TFBSs functional constraints predicted by their position weight matrix (PWM) models and highlight some intriguing exceptions. Next, we draw inspiration from Haldane's [31] and Muller's [32] genetic load model to devise a metric of TFBS variation that takes into account the loss of PWM match score associated with a mutation and makes it possible to investigate per-instance TFBS functional constraints. Finally, we take advantage of per-individual binding maps for a human transcription factor (CTCF) to highlight the 'buffering' of genetic variation at TFBSs at the level of binding, particularly in evolutionarily conserved regions.

## Results

We aim to analyze TFBS functional constraints using the binding data generated by the ENCODE, modENCODE and published sources. Prior to these global analyses, however, we first examined the relationship between binding sites' match to consensus, their conservation and variation using three well-characterized *Drosophila *TFs, Twist (Twi), Biniou (Bin) and Tinman (Tin), which have large numbers of TFBSs whose general occupancy is predictive of specific spatio-temporal activity [2]. The discovered PWMs for these TFs from both *in vitro *and *in vivo *studies are good predictors for their binding [2] and their binding sites show an appreciable level of variation, presumably much of which is deleterious but not lethal. For these TFs, 24 to 28% of the bound sites overlapped with SNPs identified by the DGRP [22] in 162 isogenic lines of *Drosophila melanogaster *(hereafter we refer to variation across these lines as 'individual variation'). As expected, variation at the same sequences detected outside of TF-bound regions (that is, at potentially random motif matches) was even higher, with 35% of them containing known SNPs (Fisher test, *P *< 1e-50 compared to the bound sites).

**Figure 1:**
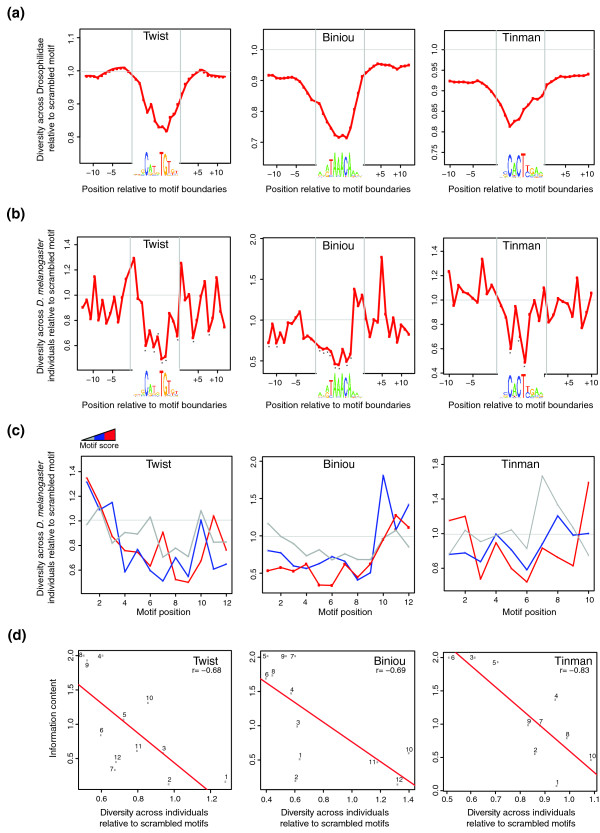
**Position-wise variation properties of three well-characterized developmental TFs from *Drosophila melanogaster***. **(a) **Interspecies diversity at bound motif positions and motif flanks. Diversity is expressed as 1-phastcons scores [64] per position across 15 insect species normalized to these scores for the scrambled versions of the same motifs detected within the respective TF-bound regions. TF 'binding logo' representations of motif PWMs are shown below each plot. **(b) **Within-species diversity at bound motif positions and motif flanks, expressed as genetic diversity (*D*) [78] per position across 162 isogenic lines of *D. melanogaster *from the DGRP normalized to the same metric for the scrambled versions of the motifs detected within the respective TF-bound regions. Asterisks indicate positions showing significantly reduced variation compared to the scrambled motifs (relative diversity <1; permutation test *P *< 5e-3). TF 'binding logo' representations of motif PWMs are shown below each plot. The non-normalized versions of the same plots, including both TF-bound and all instances of these motifs and their scrambled versions, are shown in Figure S1 in Additional file 1. **(c) **Within-species diversity per motif position across the three score ranges labeled grey to red in the increasing order: weak (Twi and Tin, 3 to 5; Bin, 5 to 8), medium (Twi and Tin, 5 to 7; Bin, 8 to 10) and strong (Twi and Tin, >7; Bin, >10). **(d) **Inverse correlation between individual variation at motif positions (x-axis) and positional information content according to motifs' PWM (y-axis). Variation is expressed in the same terms as in (b). Numbers beside the dots indicate motif positions; *r *is the Pearson's correlation coefficients for each TF. The same plots for cross-species variation are shown in Figure S2 in Additional file 1.

Focusing on the TF-bound instances of Twi, Bin and Tin motifs, we first analyzed sequence variation at each motif position across 12 *Drosophila *species (Figure 1a) and across *D. melanogaster *individuals (Figure 1b). As expected, TF-bound motifs both are conserved across evolutionary distance and show depressed levels of variation across individuals compared to either their respective flanking regions (Figure 1a,b), reshuffled motifs, unbound motifs or the third bases of Gly codons considered to be evolutionarily neutral (Figure S1A in Additional file 1). Based on these observations, we conclude that the quality and genetic diversity of the DGRP make it suitable for global analyses of TFBS variation and these data are unlikely to elicit a prohibitive bias.

PWMs are an established way of representing the sequence preferences of TFBSs, with PWM match scores reflecting the similarity of a given sequence to the hypothetical 'ideal' binding site for a given TF [33]. To study the relationship between PWM scores and variation, we compared the variation properties of Twi, Bin and Tin motifs at three score ranges ('strong', 'medium' and 'weak' scoring). Weaker (that is, potentially 'less optimal') motifs generally showed higher levels of individual variation (Figure 1c), as further confirmed using only the strongest scoring sites from each bound region to reduce the contribution of non-functional motif matches (Figure S1B in Additional file 1). This result is consistent with the expectation that selection would predominantly work towards increasing TFBSs' match to consensus [34]. We revisit this question more formally later in the study.

As well as looking across the entire PWM, we can consider each motif position in turn. Consistent with previous findings for other TFs in yeast [35] and *Drosophila *[36], cross-species variation at Twi, Bin and Tin motif positions strongly anti-correlated with their information content (Figure 1a; Figure S2 in Additional file 1). Variation across individuals also anti-correlated with positional information content (Figure 1d), confirming the general link between evolutionary conservation and population diversity [37]. There are, however, some interesting exceptions. For example, positions 6, 7 and 12 of the Twi motif are less varied in the population than would be expected from their information content (Figure 1d, left panel). These positions correspond to the 'spacer' region of the CANNTG E-box consensus motif recruiting basic helix-loop-helix (bHLH) proteins, for which specific sequence preferences were documented depending on specific dimerization partners [38]. Similarly, we found the first two positions of the Bin motif to be highly constrained despite their very low information content (Figure 1d, middle panel), suggesting that these positions may also be subject to specific restrictions depending on the *cis-*regulatory context of each motif instance. From this analysis we conclude that PWMs that have a strong correlation between information content and cross-species conservation are likely to be good descriptors of TF sequence binding preferences in a population context.

**Figure 2:**
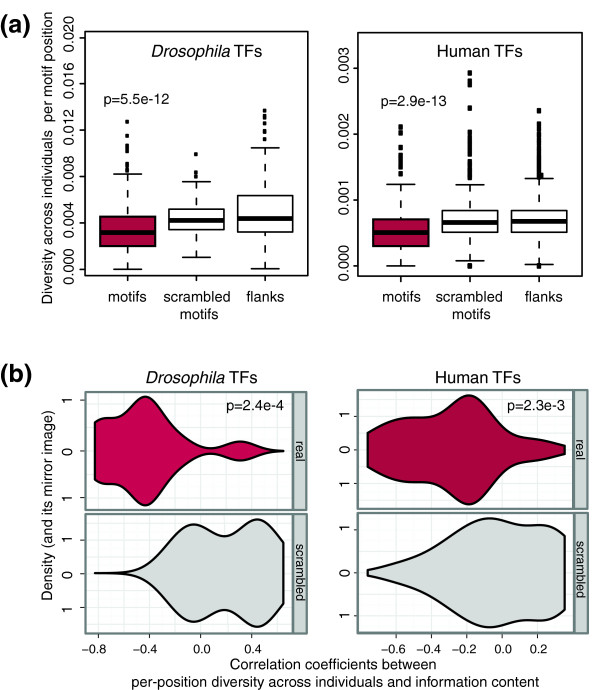
**Individual variation of the binding sites for 15 *Drosophila *and 36 human TFs selected for this study**. **(a) **Distributions of position-wise diversity at motif positions (red), scrambled motifs and motif flanks at the TF-bound regions of *Drosophila *(left panel) and human (right) TFs; *P*-values are from Kruskal-Wallis non-parametric significance tests. **(b) **Violin plots (a combination of boxplots and two mirror-image kernel density plots) showing the correlation between individual variation and information content per motif position for the bound instances of *Drosophila *(left) and human (right) TFs included in this study (top, red) and their scrambled versions detected within the same bound regions (bottom, grey); *P*-values are from Wilcoxon two-sample non-parametric significance tests.

We now turn to the human (ENCODE [23]) and *Drosophila *datasets (combined from modENCODE and other studies [2,24-30]), selecting for analysis those TFs for which position-wise conservation across species generally correlated with PWM information content. This initial filtering was done to ensure that PWMs included in the analysis reflected the global sequence constraints of these TFs' binding sites and could therefore be used to compare such constraints across TFBS instances, as presented below. Additional filtering criteria were used to ensure sufficient statistical power (in particular with respect to the total number of sites showing variation) and specificity of the analysis, resulting in the final dataset of 15 *Drosophila *and 36 human motifs (see Materials and methods and Supplementary note on TF selection in Additional file 1 for details). As before, we used DGRP data [22] to assess individual variation at *Drosophila *TFBSs, while for the humans we used Central European (CEU) genotypes sequenced as part of the 1000 Genomes Pilot Project [21] (using a Yoruban population instead of CEU yielded consistent results; not shown). Similar to our findings for the three *Drosophila *TFs, we observed reduced levels of individual variation at functional binding sites compared to reshuffled motif matches and flanking regions for other *Drosophila *factors as well as human TFs (Figure 2a). Notably, the significance of this effect was similarly high in *Drosophila *and humans, despite the fact that the SNP frequency differed approximately 11-fold (2.9% versus 0.25%, respectively), as closely reflected by the 7.5-fold difference in the number of varying TFBSs. This is consistent with the overall differences in the total number of SNPs detected in these two species, likely resulting from their different ancestral effective population sizes [39]. We also observed a significant anti-correlation between variation frequency at motif positions and their information content in both species (Figure 2b).

**Figure 3:**
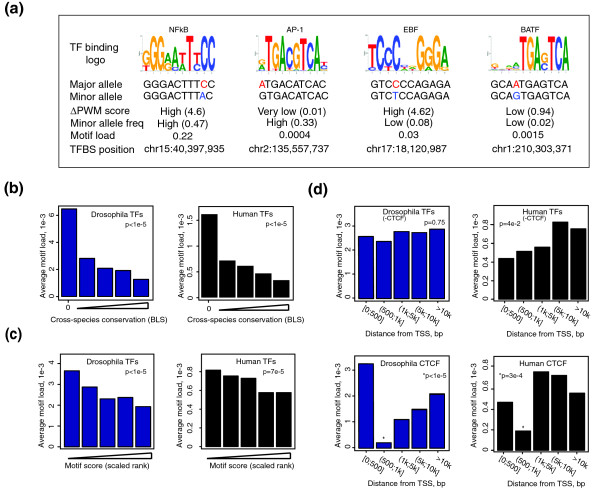
**Motif mutational load of *Drosophila *and human TFBSs located within different genomic contexts**. **(a) **Examples of mutational load values for individual instances of four human TFs (ranging from high to very low) showing different combinations of parameters that are combined in this metric: the reduction of PWM match scores at the minor allele ('ΔPWM score') and the number of genotypes within the mutation in the population (minor allele frequency (MAF)). **(b) **Relationship between phylogenetic conservation and motif mutational load for *D. melanogaster *(left) and human (right) TFs included in this study. Conservation is expressed as per-instance branch length scores (BLSs) for each instance computed against the phylogenetic tree of 12 *Drosophila *species. The average load for *D. melanogaster-*specific sites (BLS = 0) is shown separately as these have an exceptionally high motif load. **(c) **Relationship between motif stringency and motif load in *Drosophila *(left) and humans (right). Motif stringency is expressed as scaled ranked PWM scores grouped into five incremental ranges of equal size (left to right), with average motif load shown for each range. **(d) **Relationship between distance from transcription start site (TSS) and motif load in *Drosophila *(left) and humans (right) for all analyzed TFs excluding CTCF (top) and for CTCF alone (bottom), with average motif load shown for each distance range. (b-d) Average motif load is computed excluding a single maximum value to reduce the impact of outliers. The *P*-values are from permutation tests, in which permutations are performed separately for each TF and combined into a single statistic as described in Materials and methods.

So far we have been aggregating TFBSs position-by-position, which limits the scope of questions that could be addressed using these data. This has prompted us to devise a constraint metric that could be computed for individual motif instances and compared between heterologous TFBS subsets defined on the basis of their biological properties. The results presented above confirm the expected model that the deleterious effect of TFBS variation depends on how much it perturbs the motif consensus. Therefore, we proposed to express the deleterious effect of TFBS mutations in terms of 'mutational load', a known population genetics metric that combines the frequency of mutation with predicted phenotypic consequences that it causes [31,32] (see Materials and methods for details). We adapted this metric to use the reduction in PWM score associated with a mutation as a crude but computable measure of such phenotypic consequences. For example, the load of a motif instance for which no variation is observed equals zero, while the load of a motif instance with a common mutation mapping to it that results in a severe loss of PWM match score is close to 0.5 (see Figure 3a for real-life examples). As would be expected for a metric quantifying deleterious effects, motif load showed a monotonic decreasing distribution in both flies and humans (Figure S3 in Additional file 1).

We do not assume that TFBS load at a given site reduces an individual's biological fitness. Rather, we argue that binding sites that tolerate a higher load are less functionally constrained. This approach, although undoubtedly a crude one, makes it possible to consistently estimate TFBS constraints for different TFs and even different organisms and ask why TFBS mutations are tolerated differently in different contexts. Conceptual and statistical considerations associated with TFBS load are discussed at length in Materials and methods; here we will only outline several major points. First, since binding events limited to minor alleles are likely to be overlooked by a single-genome ChIP analysis, we compute the decrease in PWM match score relative to the major and not the highest-scoring allele as in the 'classic' genetic load metric. In addition, since we focus on the deleterious effects of variation, we have assumed that mutations yielding increased PWM match scores have a load of zero. We avoided the use of negative load values for these 'gain-of-score' mutations, as it is possible that such mutations will often be near-neutral, while in some cases they may even be deleterious.

Most of the analyzed TFBSs have no detected variation, in particular in human, and therefore a zero load. This affects the statistical power, making it challenging to examine many TFs one-by-one. However, analyzing the data globally for all included TFs in each organism has allowed us to identify a number of significant trends, as presented below. Technically, the high proportion of sites with no detected variation also leads to a considerable zero-inflation of TFBS load distributions, which violates the assumptions of conventional significance tests. Therefore, instead we estimate significance by using permutation tests, as further described in Materials and methods. For the same reason, we also chose to present average (more precisely, trimmed mean) TFBS load values in many comparative analyses as a metric that reflects both the frequency of variation (that is, zero versus non-zero load) and the intensity of its effect (that is, the distribution of non-zero load).

We first asked whether motif load would be able to detect the expected link between evolutionary and individual variation. We used a published metric, branch length score (BLS) [40], to characterize the evolutionary conservation of a motif instance. This metric utilizes both a PWM-based model of the conservation of bases and allows for motif movement. Reassuringly, mutational load correlated with BLS in both species, with evolutionarily non-conserved motifs (BLS = 0) showing by far the highest degree of variation in the population (Figure 3b). At the same time, approximately 40% of human and fly TFBSs with an appreciable load (L > 5e-3) still mapped to reasonably conserved sites (BLS > 0.2, approximately 50% percentile in both organisms), demonstrating that score-reducing mutations at evolutionarily preserved sequences can be tolerated in these populations.

Earlier in the study we have shown evidence that 'weaker' motifs (that is, those with a poorer PWM match) are more prone to variation, suggesting that they are less functionally constrained. Weaker sites have many more possible variants with similar match scores, while mutations at stronger sites are less likely to preserve their match. Motif load is based on the decrease in PWM score associated with mutations and not sequence variation *per se *and is therefore more 'protected' from this bias. Using this metric, we confirmed our original findings, suggesting that TFBSs with higher PWM scores are generally more functionally constrained compared to 'weaker' sites (Figure 3c). The fraction of detected sites mapping to bound regions remained similar across the whole analyzed score range, suggesting that this relationship is unlikely to be an artifact of higher false-positive rates at 'weaker' sites (Figure S4A in Additional file 1). This global observation, however, does not rule out the possibility that a weaker match at some sites is specifically preserved to ensure dose-specific TF binding. This may be the case, for example, for *Drosophila *Bric-à-brac motifs, which exhibited no correlation between motif load and PWM score (Figure S4B in Additional file 1), consistent with the known dosage-dependent function of Bric-à-brac in embryo patterning [41].

We then used motif load to address whether TFBSs proximal to transcription start sites (TSSs) are more constrained compared to more distant regulatory regions. We found this to be the case in human, but not *Drosophila *(Figure 3d; see Discussion). CTCF binding sites in both species were a notable exception, tolerating the lowest mutational load at locations 500 bp to 1 kb from TSSs, but not closer to the TSS (Figure 3d, bottom panel), suggesting that the putative role of CTCF in establishing chromatin domains [42] is particularly important in proximity of gene promoters.

**Figure 4:**
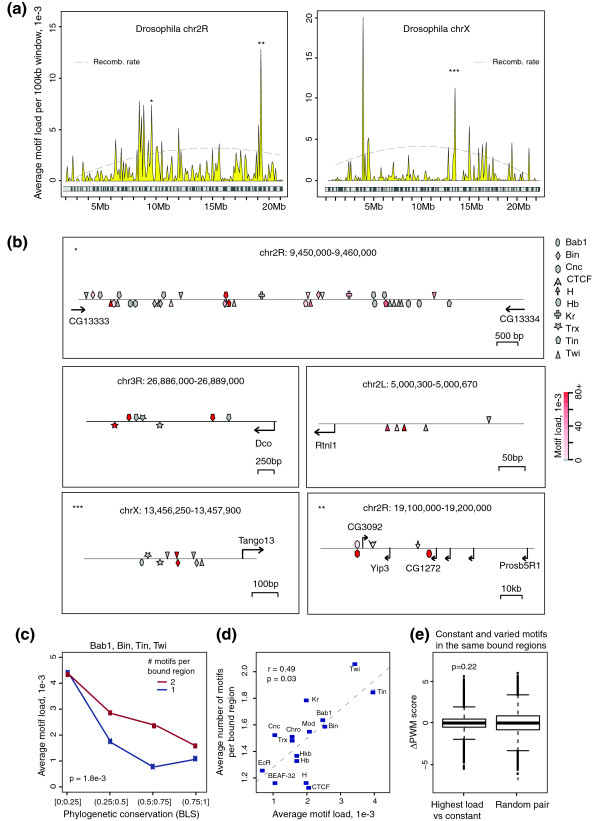
**Evidence for the 'buffering' of deleterious TFBS variation by neighboring homotypic motifs in *Drosophila***. **(a) **Distributions of average motif load per 100 kb window along *Drosophila *chromosome 2R and chromosome × (yellow; see Figure S5 in Additional file 1 for other chromosomes). Recombination rate distributions along the chromosomes (dashed lines) are from [22] (and are near-identical to an earlier analysis [43]); note that there is no apparent correlation between these two parameters. Regions of high average motif load marked with asterisks are further examined in (b). Average motif load is computed excluding a single maximum value to reduce the impact of outliers. **(b) **Examples of motif arrangement at regions that fall within 100 kb windows having high average motif load (L >5e-3). Motifs with no detected deleterious variation (L = 0) are colored grey, and those with non-zero load pink (low load) to red (high load). Asterisks refer to similarly labeled peaks from (a). Note that most high-load motifs found in these regions have additional motifs for the same TF in their proximity. **(c) **Distributions of average load across ranges of phylogenetic conservation for motifs with a single match within a bound region ('singletons', blue) versus those found in pairs ('duplets', red). For equivalent comparison, a random motif out of the duplet was chosen for each bound region and the process was repeated 100 times. Results are shown for the four TFs for which appreciable differences between 'singletons' and 'duplets' were detected. Phylogenetic conservation is expressed in terms of branch length score (BLS) ranges, similarly to Figure 2b. The *P*-value is from a permutation test for the sum of average load differences for each range between 'singleton' and 'duplet' motifs. Average load was computed excluding a single maximum value. **(d) **Relationship between the average load per TF and the average number of motifs per bound region. Average load was computed excluding a single maximum value; *r *is Pearson's correlation coefficient and the *P*-value is from the correlation test. **(e) **The difference in motif score between motif pairs mapping to the same bound regions: the one with the highest load versus one with a zero load ('constant'; left) or in random pairs (right). These results suggest that the major alleles of motifs with a high load are generally not 'weaker' than their non-varying neighbors (the *P*-value is from the Wilcoxon test).

We then considered the genome-wide properties of the mutational load metric. Recombination rates are distributed unevenly along *Drosophila *chromosomes (Figure 4a, dashed lines) [22,43]; however, we did not observe an association between the TFBS load and local recombination rates (Figure 4a; Figure S5 in Additional file 1). Rather, the analysis of selected 'high-load hotspots' (average load per 100 kb window >5e-3) revealed regions in which motifs with deleterious variation mapped in close proximity to other motifs for the same TF (see Figure 4b for examples). This suggested that TFBS mutations may be partially 'buffered' by neighboring motifs. Consistent with this model, we found that motifs for at least four *Drosophila *TFs tolerated a significantly lower load when present as 'singletons' compared to sites with two motifs (Figure 4c), particularly for evolutionarily conserved instances. Interestingly, TFs whose binding sites had a higher mean load generally had more motifs per ChIP region (Figure 4d), raising the possibility that a higher number of motifs may allow a TF to tolerate a higher load. The PWM scores of variable motifs were similar to those of 'constant' motifs in their proximity (Figure 4e); it is unlikely, therefore, that these variable motifs are non-functional *a priori*.

**Figure 5:**
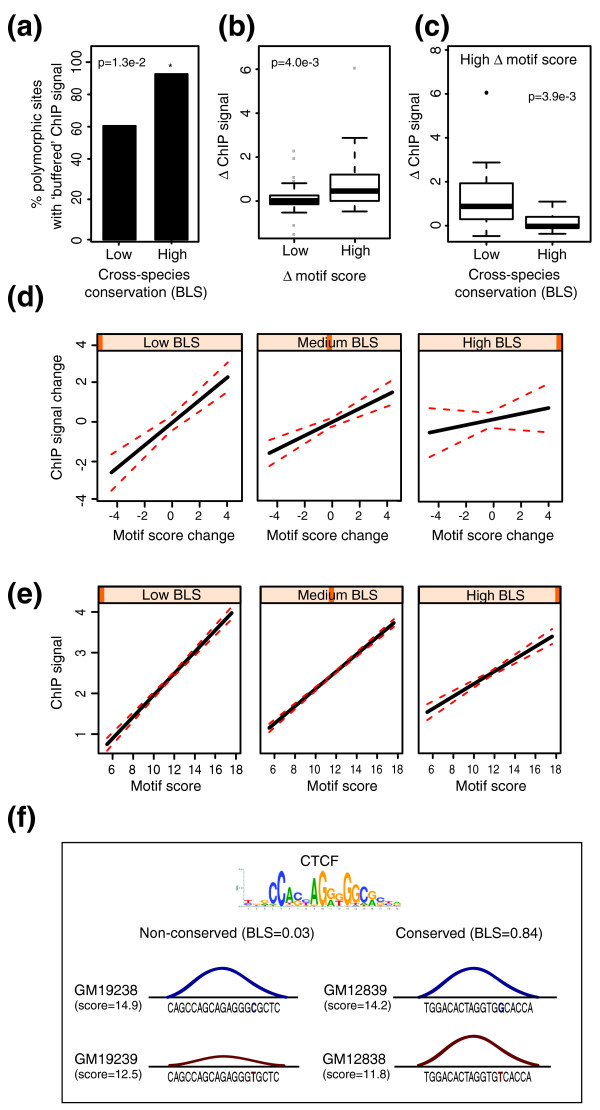
**Evidence for the 'buffering' of variation at conserved CTCF binding sites**. **(a) **Proportion of homozygous polymorphic CTCF binding sites with 'buffered' levels of ChIP signal depending on the sites' evolutionary conservation (less conserved, BLS <0.5; more conserved, BLS ≥0.5). Sites at which the minor variant retained at least two-thirds of the major variant's signal were considered as 'buffered'. The *P*-value is from the Fisher test. Major and minor variants were defined on the basis of the global allele frequency data from [75,76]. **(b) **Differences in the CTCF binding signal (Δ ChIP signal) at homozygous polymorphic sites that show either 'low' (left) or 'high' (right) disparity in absolute motif match scores (Δ motif score) between the variants (<1 or >1, respectively). The ChIP signals are sign-adjusted relative to the direction of PWM score change. Site-specific signals from multiple individuals with the same genotype, where available, were summarized by mean. The *P*-value is from the Wilcoxon test. **(c) **Genotype-specific differences in the CTCF ChIP signal across individuals between homozygous polymorphic sites with appreciable differences in absolute PWM match scores (Δ motif score >1) at less conserved (BLS <0.5, left) and more conserved (BLS >0.5, right) CTCF motifs. The ChIP signals are sign-adjusted relative to the direction of PWM score change. Site-specific signals from multiple individuals with the same variant, where available, were summarized by mean. The *P*-value is from the Wilcoxon test. **(d) **An interaction linear model showing that interspecies motif conservation (expressed by branch length scores) reduces the effect of motif mutations on CTCF binding. Shown are the effect plots predicting the relationship between the change of PWM score (at the minor versus the major variant) and the change of the associated ChIP signal at three hypothetical levels of evolutionary conservation: BLS = 0 (low; left); BLS = 0.5 (medium; middle); and BLS = 1 (high; right). Major and minor variants were defined on the basis of the global allele frequency data from [75,76]. **(e) **An interaction linear model showing that interspecies motif conservation (BLS) reduces the effect of motif stringency on the binding signal. Shown are the effect plots predicting the relationship between motif scores and ranked ChIP signal at three hypothetical conservation levels: BLS = 0 (low; left); BLS = 0.5 (medium; middle); and BLS = 1 (high; right). **(f) **A schematic illustrating the observed effect of binding site mutations on CTCF binding signal at two polymorphic CTCF sites - one poorly conserved (BLS = 0.03, left) and one highly conserved (BLS = 0.84, right) - that have similar motif match scores (14.9 and 14.2, respectively). Sequences of higher- (top) and lower-scoring alleles (bottom) are shown on the figure. Mutations resulting in a similar loss of score (down to 12.5 and 11.8, respectively) resulted in a 53% loss of CTCF binding signal at the non-conserved site (left, compare the amplitudes of top (blue) to bottom (red) curves), in contrast to a mere 6% at the conserved site (right).

To gain further insight into the functional effects of TFBS mutations, we used a dataset that mapped human CTCF binding sites across four individuals from [16] (see Materials and methods for more details). TFBS mutations detected in this dataset often did not result in a significant loss of binding, with approximately 75% of mutated sites retaining at least two-thirds of the binding signal. This was particularly prominent at conserved sites (BLS >0.5), 90% of which showed this 'buffering' effect (Figure 5a). To address whether buffering could be explained solely by the flexibility of CTCF sequence preferences, we analyzed between-allele differences in the PWM score at polymorphic binding sites. As expected, globally CTCF binding signal correlated with the PWM score of the underlying motifs (Figure S6A in Additional file 1). Consistent with this, alleles with minor differences in PWM match generally had little effect on the binding signal compared to sites with larger PWM score changes (Figure 5b), suggesting that the PWM model adequately describes the functional constraints of CTCF binding sites. At the same time, we found that CTCF binding signals could be maintained even in those cases where mutations resulted in significant changes of PWM score, particularly at evolutionarily conserved sites (Figure 5c). A linear interaction model confirmed that the effect of motif mutations on CTCF binding was significantly reduced with increasing conservation (Figure 5d; interaction term *P *= 2.9e-2). These effects were not due to the presence of additional CTCF motifs (as 96% of bound regions contained only a single motif), while differences between more and less conserved sites could not be explained away by differences in the PWM scores of their major alleles (not shown). A CTCF dataset from three additional individuals generated by a different laboratory [44] yielded consistent conclusions (Figure S6B-D in Additional file 1), suggesting that our observations were not due to overfitting.

Taken together, CTCF binding data for multiple individuals show that mutations can be buffered to maintain the levels of binding signal, particularly at highly conserved sites, and this effect cannot be explained solely by the flexibility of CTCF's sequence consensus. We asked whether mechanisms potentially accountable for such buffering would also affect the relationship between sequence and binding in the absence of mutations. Training an interaction linear model across the whole set of mapped CTCF binding sites revealed that conservation consistently weakens the relationship between PWM score and the binding intensity (*P *= 1.9e-7; Figure 5e). Thus, CTCF binding to evolutionarily conserved sites may generally have a reduced dependence on sequence.

## Discussion

Deciphering the *cis*-regulatory 'logic' of gene regulation is one of the biggest challenges genomics faces today. Understanding the functional constraints of regulatory elements across species has been the focus of much 'evo-devo' research, leading to many exciting insights, such as the preservation of CRM function without a base-to-base preservation of sequence [9-11] and the impact of protein-protein interactions [45]. Variation across individuals presents a snapshot of 'evolution in action', giving access to potentially suboptimal alleles without having to resort to artificial perturbation, and are a promising resource for population functional genomics studies as well as more formal association analyses. Such 'pop-fun' approaches will complement the insights obtained from 'evo-devo' studies.

Here we have used three different approaches to investigate TFBS functional constraints based on variation data. In the first one, using position-by-position comparisons, we have found that variability at TFBS positions generally correlates with information content, consistent with previous findings based on cross-species comparisons in *Drosophila *and human for other TFs [35,36] and population studies in yeast [18]. It should be noted that the majority of PWMs used in this study have been derived from comparing the sequences across all binding sites in one genome detected by genome-wide ChIP studies. Variation analyses look at sequence diversity in a different 'dimension': that is, across individuals at a particular point in the genome for each given binding site. That these two dimensions generally correlate with each other (and often also with *in vitro *biochemical data such as SELEX and protein binding microarrays [46,47]) has been a reassuring confirmation of the general validity of PWM models to describe the sequence 'code' for the analyzed TFs. This, in turn, is an important prerequisite for using PWM scores to compute TFBS mutational load, a per-instance metric that combines the penetrance of a motif mutation with the loss of the PWM match it causes.

*Cis*-regulatory variation is accountable for serious deleterious effects, and yet it is common [14,20]. Understanding TFBS functional constraints is therefore interesting for at least two reasons. First, it may shed light on the regulatory architecture of the genomes. For example, our finding that CTCF motifs tolerate the lowest load a short distance away from TSSs underlines the importance of chromatin architecture at the distal ends of promoter regions. In addition, TFBS constraints are indicators of how the system deals with noise in *cis*-regulatory networks, and the variation analyses presented here support such phenomena as homotypic redundancy [48]. Interestingly, it was previously shown that homotypic clustering does not affect *Drosophila *TFBS turnover rate in the phylogenetic context [36], but the dynamics of selection inside a population need not correspond to that observed between species. For example, retaining multiple instances of neighboring homotypic sites in a given species may in itself bear the selective advantage to provide robust buffering to variation and other perturbations.

Genetic load, the concept that lies at the foundation of our constraint metric, was initially put forward by J Haldane [31] and HJ Muller [32], primarily in the context of the debate on hard versus soft selection. Here, however, we use this metric outside of such context and fully acknowledge that this is a crude, albeit computable parameter. We do not imply that a high TFBS load weakens the fitness of the individual bearing it, as would be the case in the 'classic' application of this concept. Rather, we take advantage of this concept to inquire why this probably does not occur - that is, why mutations at TFBSs are tolerated differently in different genomic contexts, likely without causing a significant reduction of an individual's fitness.

There is no doubt that mutational load is an imperfect metric. More sophisticated models linking fitness to the PWM score have been developed for cross-species phylogenetic analyses [49,50] and their adaptation to population studies, although likely not straightforward, would be interesting to explore in the future. In addition, we know that the basic assumption of PWM models - that the frequency of nucleotide N at motif position K is proportionate to its positive impact on the binding affinity - does not always hold and even when it does, the amplitude of this effect may not be fully consistent across the TFs. Differences between motif sequences at different genomic locations may reflect TFBS optimization for a specific context rather than a lack of constraint. It was shown, for example, that differences at just two positions of the glucocorticoid receptor motif affect the choice of binding partners [51], while different *k*-mers of the apparently degenerate RACRYNNNNNACG motif in yeast are associated with the regulatory regions of genes with different functions [52]. It is possible, therefore, that some mutations resulting in a loss of PWM match are, in fact, beneficial rather than deleterious and may be indicative of positive selection that was recently shown to occur at a fraction of *Drosophila *TFBSs by He *et al*. [12]. However, in line with the assumption of He *et al*., we believe that the predominant direction of positive selection would be towards increasing PWM scores, and such mutations will have a zero load according to our definition.

These limitations, however, are universal for the problem of modeling functional constraints based on sequence alone. The predictive power of PWMs is probably comparable with our ability to predict the impact of mutations on RNA and protein structure. The rapidly increasing bulk of genotyping data will increase the statistical power of these analyses, but only experimental validation of the effects of TFBS mutations can give a definitive answer. This is why direct analyses of TF binding across individuals hold much promise. Using multi-individual CTCF binding maps [16,44], it was reassuring to confirm that the loss of CTCF binding associated with a TFBS mutation is generally proportionate to its impact on motif PWM match. But perhaps more importantly, using these data has allowed us to observe that this relationship does not always hold, suggesting that variation at many sites, and in particular the most evolutionarily conserved ones, can be efficiently buffered at the binding level. We do not know the exact nature of these buffering mechanisms, and whether their prevalence at highly conserved sites is evolutionarily driven or is merely a side effect of the increasing complexity of regulatory networks [53,54]. It can be expected that such buffering effects would be, at least in part, due to interactions with heterologous proteins. Given the multifaceted functions of CTCF, it is very likely that such interactions will involve different partners, depending on specific regulatory context. Studies of more 'specialized' TFs may therefore be more appropriate to address these questions. For example, analyses of individual variation at human NFκB [15] and yeast Ste12 [17] pinpointed candidate interaction partners that affect the binding in the absence of mutations at the analyzed TF's own binding sites. We attempted to use the NFκB data to ask the reverse question, that is, look for factors that may help maintain the binding when mutations at conserved TFBSs are present; unfortunately, the number of such cases was extremely low, prohibiting this analysis. It is possible that mutations at conserved NFκB sites are poorly tolerated, implying that they are less efficiently 'buffered'. However, studies involving a larger number of individuals and/or using organisms with higher variation rates, such as *Drosophila*, will be required to adequately address this question.

Theoretically, TFBS mutations can be buffered at many different levels - starting from the motif itself that may 'absorb' a number of mutations due to a permissive consensus, to the level of CRMs (for example, homotypic motifs and protein interaction partners), *cis-*regulated genes (involving possible 'backup' by shadow enhancers [55]) as well as further along the regulatory network [56] - which may potentially explain the apparent redundancy that is often observed in the network architecture, both at the level of cooperative TF binding to enhancers and multiple 'cross-talking' pathways [57]. Consistent with previous observations at individual CRMs [58], our observations suggest that much variation is buffered immediately in *cis*, via the redundancy of TFBS consensus sequences, neighboring homotypic motifs or other factors preserving regulator binding (or at least the overall CRM output). If true, this model may explain two of our preliminary observations that we initially found puzzling: that the levels of tolerated load did not significantly vary depending on the functional annotation of regulated genes (not shown) and that candidate *Drosophila *enhancers with seemingly very deleterious mutations at Bin, Tin and Twi binding sites were still able to drive reporter gene expression *in vitro *(Figure S7 in Additional file 1). It is clear, however, that this phenomenon requires further investigation, perhaps drawing more input from the biology of individual TFs. Finally, it is worth noting that a number of disease-causing mutations are located in regulatory regions, and presumably are either not buffered or inappropriately buffered. A well-studied example of this is the regulatory mutations in *Pax6 *regulatory regions associated with neurodevelopmental abnormalities [59]. In addition, the majority of genome-wide association studies do not implicate a protein-coding variant [20]. To fully understand these diseases we must gain a more complete knowledge of how variation impacts regulatory function.

## Conclusions

Integrating genome-wide TF binding profiles with individual variation data in *Drosophila *and humans, we show that TFBSs are functionally constrained and yet mutations at them can be tolerated, providing evidence for possible 'buffering' effects. Beyond their direct biological implications, these results highlight the potential of integrating functional genomics and population genetics approaches for understanding *cis-*regulatory function.

## Materials and methods

### Data sources and basic analysis

Motif discovery data were from the modENCODE and ENCODE repositories [23,24,60,61], with the exceptions of Bin, Tin and Twi that were from Zinzen *et al*. [2]. *Drosophila *ChIP data were from Zinzen *et al*., modENCODE and other published sources [2,24-30]; human ChIP data were from ENCODE [23] (see Tables S1 and S2 in Additional file 2 for details). CTCF multi-individual data were from [16,44]. EPO alignments for 12 mammals were from Ensembl [62,63]; phastcons scores [64] and multiz alignments for 12 *Drosophila *species were from Flybase [65,66]. *Drosophila *variation data were from the DGRP [22], additionally filtered as described below. Human variation data were from the 1000 Genomes Pilot Project [21]. Motif matches were detected using patser [67] (in case of overlapping matches, only the strongest-scoring motif was included) and overlaps with ChIP regions ('bound' motifs) were called using bedTools [68]. Analysis was performed in R, Python and Perl with Ensembl API.

### Filtering of DGRP data

DGRP SNPs were additionally filtered according to the following criteria: *ε *≤ 0.02 (per SNP); *p *× *ε *≤ 0.01 (per allele); coverage ≥ 3 (per allele); median coverage ≤20 (across strains); number of strains with detected homozygous alleles ≥100; number of strains with calls scored as 'heterozygous' ≤5%. The combination of these filters removed 31.3% low-confidence SNPs and increased the overlap with the SNPs detected by the *Drosophila *Population Genomics Project [69] based on a subset of the same *Drosophila *lines (not shown).

### Motif selection for the analysis

For each modENCODE and ENCODE TF, a single combination of motif and cell type was chosen based on appreciable enrichments at TF-bound versus unbound regions, the total numbers of TF-bound motifs and a correlation between per-position evolutionary conservation and information content. Motif PWM score thresholds for human TFs were determined using TFM_PVALUE (*P *= 4e-8) [70], consistent with the thresholds used in ENCODE integrative analyses [23]. For *Drosophila *TFs, thresholds were defined based on balancing the number of detected instances and motif enrichment at bound compared to unbound regions. Near-identical PWMs were removed based on Pearson correlation analyzed with STAMP [71,72]. See Supplementary note on TF selection in Additional file 2 for more detail. The properties of selected motifs are listed in Tables S1 and S2 in Additional file 2. PWMs are listed in the data/motifs.txt files at [60] and [61], respectively. The positions, sequences, PWM scores and variation properties of all TFBSs included in this study are listed in Additional file 3 (*Drosophila*) and Additional file 4 (human).

### Position-wise motif analysis

Reshuffled PWMs were generated by ten per-position permutations of the 'real' PWMs. Reshuffled motif matches were detected within the 200 bp proximity of real TF binding sites at the same PWM score thresholds as the real motifs. Position-wise variation data obtained for each permuted motif instance was then 'de-reshuffled' to match the positions of the real PWM to compute the total diversity per permuted motif position. For human motifs, the score thresholds used elsewhere in the study resulted in very low numbers of reshuffled motif instances detected near the corresponding TF binding sites. To overcome this, analyses in Figure 2 used slightly relaxed score thresholds for both real and reshuffled human motifs, adjusted such that the total number of motif instances detected with the 10 reshuffled PWMs was at least 1.5-times higher than the number of real instances for each TF.

### Branch length score

BLS calculation was reimplemented in Perl for distributed computation on an LSF compute farm according to [40], allowing for a 50 bp motif movement either way along the alignment and a drop of motif score ≤1. Branch lengths are given relative to 12 eutherian mammals or 12 *Drosophila *species, respectively. Tree lengths were computed using Ensembl API.

### TFBS mutational load

We defined motif mutational load as:

$$L=\frac{w_{0}-\sum w_{i}p_{i}}{w_{0}}$$

where *w*_0 _is the PWM score of the major allele, and *w*_i _and *p*_i _are the score and frequency of each allele, respectively. Classically, genetic load is expressed with respect to the maximum observed value (*w*_0 _= *w*_max_). However, we have instead chosen to express it relative to the major allele (*w*_0 _= *w*_maj_). The main reason for this is that, in the absence of ChIP data for each individual or isogenic line, TFBSs whose minor alleles have a higher PWM score than the major allele are subject to a significant ascertainment bias. Indeed, only TF-bound TFBS instances are included in the analysis, and we are much more likely to detect TFBSs as 'bound' when their weaker major alleles are also strong enough to ensure TF binding. Additionally, for reasons explained in the main text, we have postulated that TFBSs with stronger-scoring minor alleles have a zero load irrespective of frequency. Using the human data presented an additional challenge of interpreting heterozygous genotypes. Since the immediate phenotypic trait associated with TFBS's match to consensus (that is, TF binding) occurs in *cis*, we have taken the decision to consider each human allele separately. We did not focus exclusively on homozygous genotypes, as this approach would further reduce the statistical power of the analysis that was already limited by the low variation rates in the human genome.

### Significance testing of TFBS load

Significance testing on TFBS load data was non-trivial, as their distributions are sparse (especially in the case of human data), with the majority of TFBSs having a load of zero. In statistical terms, these data present a case of zero-inflation, in which the observed zeros are a mixture of missing data (that is, mutations that are not observed due to a limited number of available genotypes) and 'real' zeroes (mutations that never occur because their deleterious effect is prohibitively strong). To overcome this problem, we have initially used generalized additive models (gam) based on zero-inflated distributions of the response variable (ZAGA for *Drosophila *and BEINF0 for human implemented in the R package *gamlss *[73]; not shown). However, gam *P*-values may be difficult to interpret, especially when the model includes random effects [73] (in our case, the TF identity). We have therefore eventually turned to permutation tests, permuting motif load values separately for each TF to avoid bias associated with specific properties of individual factors. To test the significance of trends, we used a permutation statistic based on [74]: the dot product of the normalized data vector × and the index vector (1,..., N), where N is the length of X.

### CTCF per-individual ChIP analysis

The analysis was based on lymphoblastoid lines, for which genotypes were available from the 1000 Genomes Pilot Project [21]. We focused on the CTCF-binding data from McDaniell *et al*. [16] (Gm12892, Gm19239, Gm19238 and Gm19240) and confirmed the results using an independently generated dataset (Gm12872, Gm12873 and Gm12874) [44] processed through quantile normalization using the R/Bioconductor package *preprocessCore*. The remaining two datasets from [16] (Gm12878 and Gm12891) were excluded due to highly inconsistent overall binding score distributions. Global major allele data were from [75,76]; assuming all reference alleles as major gave consistent results (not shown). Interaction models were plotted using the R package *effects *[77]. The sequences, PWM scores and ChIP binding signals for all TFBSs included in these analyses are listed in Additional files 5 (individuals from [16]) and 6 (individuals from [44]).

## Abbreviations

Bin: Biniou; BLS: branch length score; bp: base pair; ChIP: chromatin immunoprecipitation; CRM: *cis-*regulatory module; DGRP: *Drosophila *Genetic Reference Panel; ENCODE: Encyclopedia of DNA Elements; NF: nuclear factor; PWM: position weight matrix; SNP: single-nucleotide polymorphism; TF: transcription factor; TFBS: transcription factor binding site; Tin: Tinman; TSS: transcription start site; Twi: Twist.

## Authors' contributions

MS and EB designed the study, devised the TFBS load metric and wrote the paper. MS performed *in silico *analyses with assistance from KB, GK, CG and JH. PK, MK and CG carried out motif discovery. JA performed luciferase assays. EEF co-supervised the work of JA, CG and MS and provided critical input to the entire manuscript.

## Supplementary Material

Additional file 1 **Supplementary figures S1 to S7 and Supplementary note**. Figure S1: individual variation of bound and unbound Twi, Bin and Tin motifs. Figure S2: relationship between cross-species variation and information content at Twi, Bin and Tin motifs. Figure S3: general distributions of TFBS load in *Drosophila *and human. Figure S4: additional information for the analysis of TFBS load relative to PWM match score. Figure S5: distributions of TFBS load along *Drosophila *chromosome arms. Figure S6: additional information on the per-individual analysis of CTCF binding. Figure S7: naturally occurring mutations at mesodermal TFBSs do not affect *in vitro *CRM activity. Supplementary note: selection of TF binding motifs for the analysis. Click here for file

Additional file 2 **Tables S1 and S2**. A two-sheet Excel file listing the properties of *Drosophila *(Table S1) and human (Table S2) TFs included in this study. Click here for file

Additional file 3 ***Drosophila *TFBS instances included in this study and their variation properties**. A plain text table listing the position, sequence, PWM match score, branch length score (BLS), mutational load (L), distance from the nearest TSS and, when detected, the count and PWM score of the alternative allele for *Drosophila *TFBSs included in this study. Click here for file

Additional file 4 **Human TFBS instances included in this study and their variation properties**. A plain text table listing the position, sequence, PWM match score, branch length score (BLS), mutational load (L), distance from the nearest TSS and, when detected, the count and PWM score of the alternative allele for human TFBSs included in this study. Click here for file

Additional file 5 **CTCF binding and TFBS variation properties for four individuals from McDaniell *et al***. A plain text table listing the position, sequence properties and ChIP binding signals at CTCF binding sites with detected variation in four individuals from [16]. Click here for file

Additional file 6 **CTCF binding and TFBS variation properties for three individuals from Maurano *et al***. A plain text table listing the position, sequence properties and ChIP binding signals at CTCF binding sites with detected variation in three individuals from [44]. Click here for file
