# Supplementary material for: Analysis of variation at transcription factor binding sites in Drosophila and humans
Source: Genome Biol. 2012 Sep 5;13(9):R49. doi: 10.1186/gb-2012-13-9-r49 (PMC3491393; doi:10.1186/gb-2012-13-9-r49)
Supplement: Additional file 1 — Supplementary figures S1 to S7 and Supplementary note. Figure S1: individual variation of bound and unbound Twi, Bin and Tin motifs. Figure S2: relationship between cross-species variation and information content at Twi, Bin and Tin motifs. Figure S3: general distributions of TFBS load in Drosophila and human. Figure S4: additional information for the analysis of TFBS load relative to PWM match score. Figure S5: distributions of TFBS load along Drosophila chromosome arms. Figure S6: additional information on the per-individual analysis of CTCF binding. Figure S7: naturally occurring mutations at mesodermal TFBSs do not affect in vitro CRM activity. Supplementary note: selection of TF binding motifs for the analysis. [file gb-2012-13-9-r49-S1.pdf]

Spivakov *et al.*: Analysis of variation at transcription factor binding sites in *Drosophila* and humans.

Additional file 1: Figures S1 – S7 and Supplementary note.

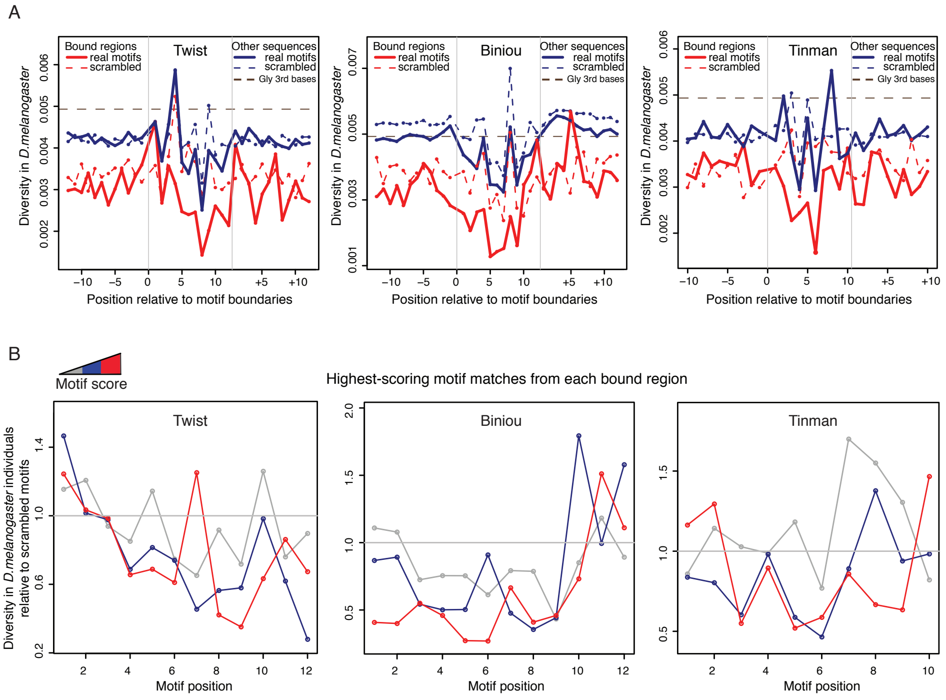

Figure S1. Individual variation of bound and unbound Twi, Bin and Tin motifs

(**A**) Non-normalised diversity values for the positions and flanks of Twi, Bin and Tin motifs (solid lines) and their 10 different scrambled versions (dashed lines) detected at TF-bound (red) and unbound (blue) regions. Diversity of the 3rd bases of Glycine codons that are considered evolutionary near neutral is shown for reference (grey dashed lines).

(**B**) A variant of analysis shown in Figure 1C with only a single highest-scoring motif match selected from each TF-bound region. Within-species diversity per motif position for each binding region shown across the three score ranges labelled grey to red in the increasing order: weak (Twi and Tin: 3–5, Bin: 5–8), medium (Twi and Tin: 5–7, Bin: 8–10) and strong (Twi and Tin: >7, Bin: >10). Similarity of these results to those shown in Figure 1C confirms that a higher diversity at 'weaker' motifs is unlikely to result solely from false-positive motif matches.

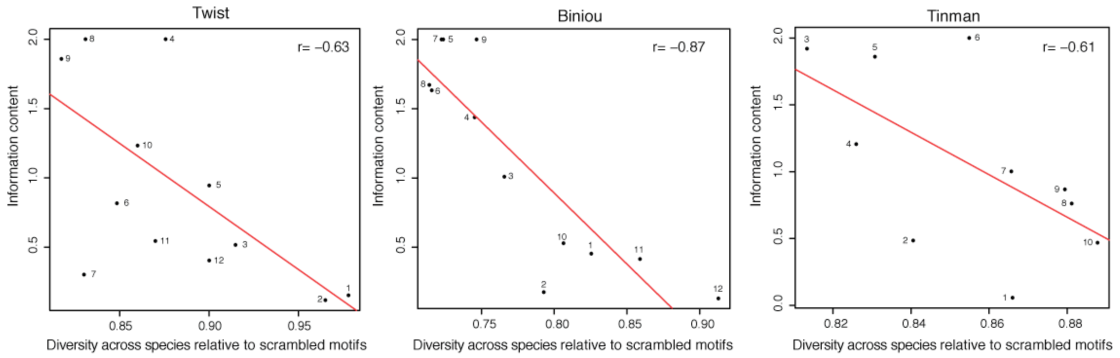

Figure S2. Relationship between cross-species variation and information content at Twi, Bin and Tin motifs

Correlation between cross-species variation at motif positions (X axis) and positional information content according to the motifs' Position Weight Matrices (Y axis). Cross-species is expressed as 1-phastcons scores across 15 insect species normalised to these scores for the scrambled versions of the same motifs detected within the respective TF-bound regions. Numbers beside the dots indicate motif positions; *r* - Pearson's correlation coefficients for each TF.

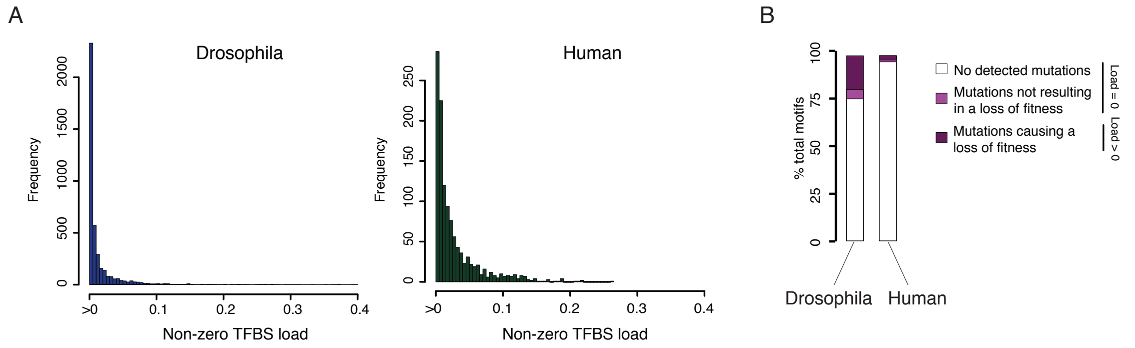

Figure S3. General distributions of TFBS load in *Drosophila* and human

(**A**) Distributions of non-zero TFBS load across *Drosophila* (left) and human (right) sites. Note that these distributions do not include sites with L=0 (i.e., with no detected mutations or none that negatively affect PWM fitness).

(**B**) Proportion of TFBSs with detected mutations that reduce (dark purple) or increase (light purple) PWM fitness compared to the major allele, and those with no detected variation (white) in *Drosophila* (left) and humans (right).

A

B

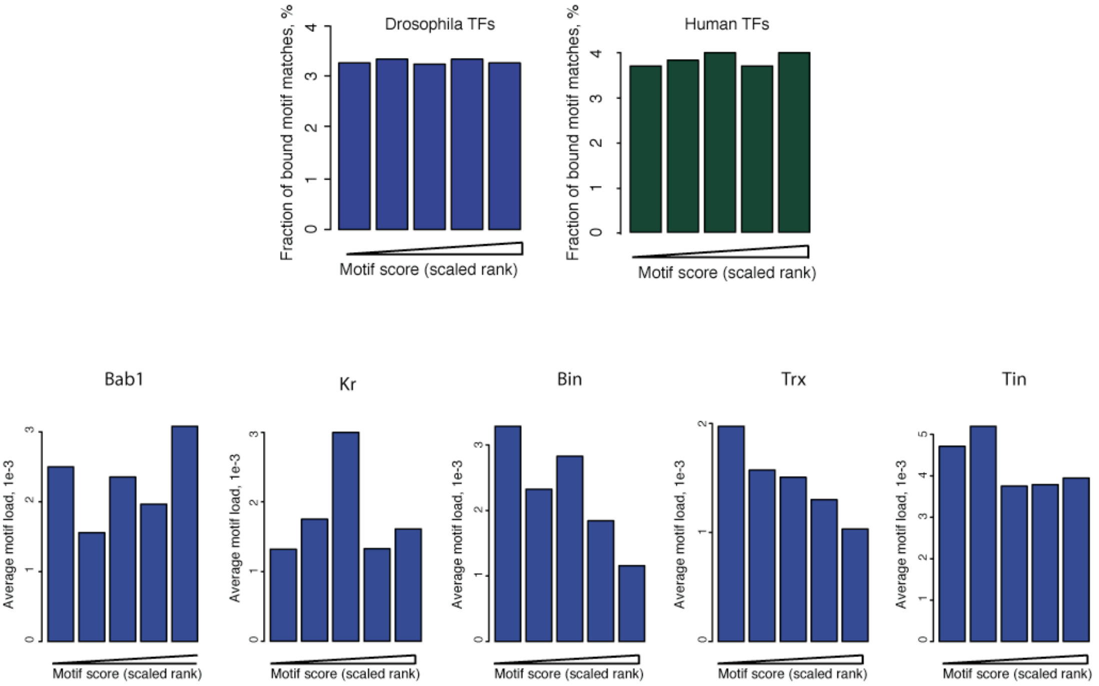

Figure S4. Additional information for the analysis of TFBS load relative to PWM match score

(**A**) Fraction of motif matches in the genome mapping to TF-bound regions at each of the scaled-ranked score ranges used in Figure 3C for *Drosophila* (left) and human (right). The fact that this fraction remains similar across the whole score range in both organisms suggests that the generally higher tolerated TFBS load at 'weaker' sites shown in Figure 3C is unlikely to be an artefact of higher false-positive rates.

(**B**) Relationship between motif score and mutational load for several *Drosophila* TFs Bric-à-brac motifs that show a similar TFBS load across the whole stringency range are shown alongside four other TFs with large numbers of detected binding sites (>850), many of which show a reduced average load at higher-scoring motifs. Motif stringency is expressed as scaled ranked PWM scores grouped into five incremental ranges of equal size (left to right), with the average (trimmed mean) motif load shown for each range.

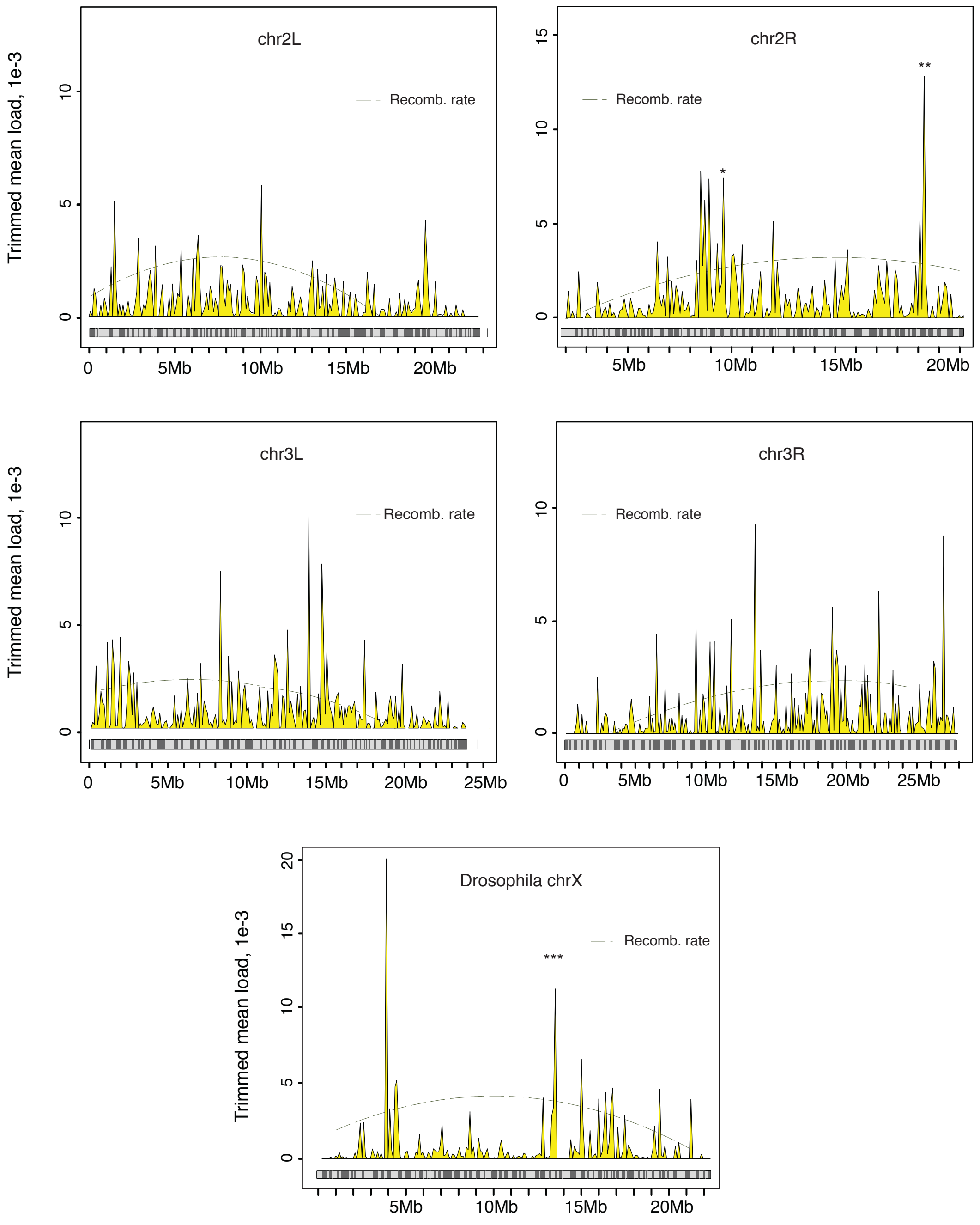

Figure S5. Distributions of TFBS load along *Drosophila* chromosome arms

Distributions of average motif load per 100kb window (yellow polygons; see Figure 4A for chr2R and chrX). Recombination rate distributions along the chromosomes (dotted lines) are from [22]; note that there is no apparent correlation between these two parameters. Average motif load is computed excluding a single maximum value to reduce the impact of outliers.

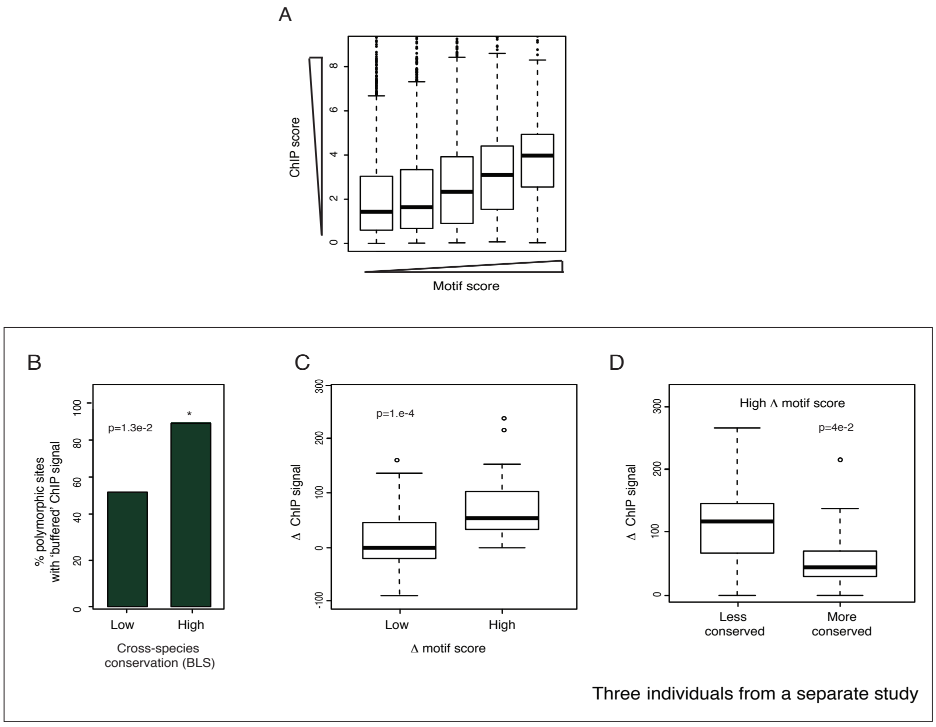

Figure S6. Additional information on the per-individual analysis of CTCF binding

(**A**) Relationship between CTCF motif score and binding intensity shown as boxplots across five incremental motif score ranges of equal size.

(**B, C, D**) Data for three individuals from a ChIP dataset for three human individuals [44] generated independently from the data used in Figure 5 ([16]). Note that the scores in [16] and [44] are not to scale. (**B**) Proportion of homozygous polymorphic CTCF binding sites with 'buffered' levels of ChIP signal depending on the sites' evolutionary conservation (less conserved: Branch Length Score < 0.5, more conserved: Branch Length Score ≥ 0.5). Sites at which the minor variant retained at least two thirds of the major variant's signal were considered as 'buffered'. The p-value is from the Fisher test. Major and minor variants were defined on the basis of the global allele frequency data from [75, 76]. (**C**) Differences in the CTCF binding signal (Δ ChIP signal) at homozygous polymorphic sites that show either 'low' (left) or 'high' (right) disparity in absolute motif match scores (Δ motif score) between the variants (< 2 or > 2, respectively). The ChIP signals are sign-adjusted relative to the direction of PWM score change. Signals from multiple individuals with the same variant, where available, are summarised by mean. The p-value is from the Wilcoxon test. (**D**) Genotype-specific differences in the CTCF ChIP signal across individuals between homozygous polymorphic sites with appreciable differences in absolute PWM match scores (Δ motif score > 1) at less conserved (Branch Length Score < 0.5, left) and more conserved (Branch Length Score > 0.5, right) CTCF motifs. The ChIP signals are sign-adjusted relative to the direction of PWM score change. Site-specific signals from multiple individuals with the same variant, where available, are summarised by mean. The p-value is from the Wilcoxon test.

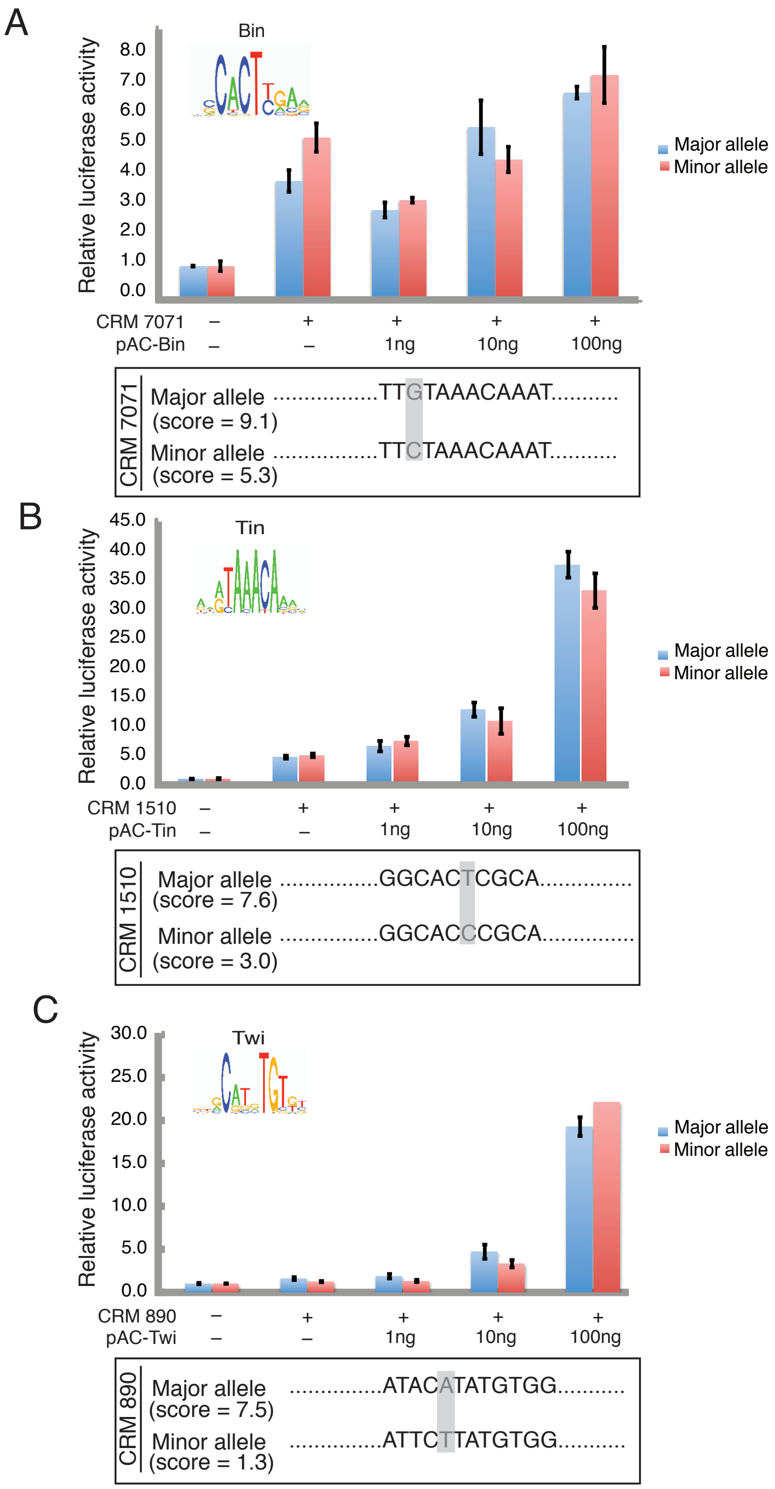

Figure S7. Naturally occurring mutations at mesodermal TFBSs do not affect *in vitro* CRM activity. See legend on the next page.

Figure S7 (previous page). Naturally occurring mutations at mesodermal TFBSs do not affect *in vitro* CRM activity

Luciferase assays on three CRMs from Zinzen et al. [2], for which the minor alleles of TFBSs mapping to them have significantly reduced PWM scores. (**A**) CRM 7071 with a segregating Biniou site. (**B**) CRM 1510 with a segregating Tinman site. (**C**) CRM 890 with a segregating Twist site. Sequences of the major and minor alleles (defined on the basis of DGRP data [22]) are shown in boxes below plots, with segregating positions highlighted in grey and PWM match scores shown in brackets. As can be seen from the plots, CRM versions with both higher-scoring and lower-scoring TFBS alleles respond similarly to activation by the different doses of the corresponding TF in luciferase assays in S2 cells. Error bars indicate standard errors of measurement from three independent experiments. TF motif logos are shown for reference inside the plots.

Supplementary note. Selection of TF binding motifs for the analysis

Selection of fly TFs

The initial set of TF binding motifs was selected from the modENCODE fly motif repository [65] filtered on the basis of enrichment at bound regions of at least 3 (motif discovery in [61] has been performed on the modENCODE ChIP data [24, 25] as well as a number of previously published datasets [26-30]). Optimized motif versions for Bin, Tin, and Twi and the corresponding binding data from [2] were added to the pool. The resulting motif dataset included 162 out of the total 312 motifs (for 49 out of 57 TFs) plus 16 additional motifs associated with Homeobox TFs. These motifs were clustered using STAMP [71, 72] based on Pearson correlation, giving clusters listed below (the tree was cut at ~99% from root). The match score thresholds used in [61] (and in the integrative modENCODE analyses [24]) often resulted in very low numbers of motifs detected at TF-bound regions. We therefore sought to reduce these thresholds to ensure that at least 150 non-overlapping TF-bound motifs are detected within at least 120 TF-bound regions, while requiring that motif enrichment at TF-bound regions does not fall below 1.8. We also required a correlation between conservation (phastcons) and information content of at least 0.3. For 15 TFs, at least one motif passed this filter. A single best combination of motif and cell/embryo type was chosen for each selected TF. The included motifs are shown in bold in the clusters below and listed in Table S1.

(su_Hw__known1, su_Hw__disc2, su_Hw__disc1, Cp190_disc6, **mod_mdg4__disc3**)
(D_disc4, jumu_disc3)
(sna_known5, bin_disc1, **bin_ef**)
(Mef2_disc1, Kr_disc4)
(Homeobox_known11, Homeobox_known53)
(gt_known1, gt_known5)
(bab1_known2, Mef2_disc2) - discarded as another motif used for bab1 and Mef2_disc2 showed enrichments >=1.8 only at very low bound-region recall values (~2%)
(Kr_known1, Kr_known7, Kr_known2, **Kr_known3**, Kr_known8, Kr_disc3, Kr_known6, Kr_known4, Kr_known5, Kr_known9)
(Homeobox_known4, Homeobox_known35, Homeobox_known21, Homeobox_disc6)
(**EcR_known1**, ttk_known1, ftz-f1_known2)
(**bab1_disc1**, Kr_disc2, Cp190_disc2)

(Homeobox_known10, Homeobox_disc10, Homeobox_known27, Homeobox_known39, **hkb_known2**)
(Cp190_disc1, CBP_disc1, h_disc4, **trx_disc1**, sens_disc1, jumu_disc1, hkb_disc2, Chro_disc2, Snr1_disc1, trx_disc2, Chro_disc5)
(z_known4, mod_mdg4__disc4)
(**cnc_disc1**, cnc_disc2)
(Homeobox_disc4, h_disc5, Hr4_disc1, Hr4_disc2)
(HP1_disc2, HP1_disc4)
(Stat92E_known1, Stat92E_known2, Stat92E_disc1)
(dl_known6, shn_known2, dl_known7)
(dl_disc1, run_disc5, jumu_disc4, dl_disc2, Chro_disc7, Cp190_disc8)
(Trl_disc1, cbt_disc1, br_disc2, cbt_disc2, HP1_disc1)
(**BEAF-32_disc1**, h_disc1, CBP_disc2, D_disc1, Homeobox_disc3, Chro_disc1, run_disc3, trem_disc1, Cp190_disc3, GATAe_disc1, BEAF-32_disc4, BEAF-32_disc6, GATAe_disc2)
(BEAF-32_disc5, CBP_disc4, Chro_disc4) - discarded as too similar to the selected BEAF-32 motifs
(**h_known1**, h_known3, h_known2, h_known4, h_disc6, NELF-B_disc1, Nelf-E_disc1, disco_disc1)
(**CTCF_known1**, CTCF_disc4, Kr_disc1, CTCF_disc1, bab1_disc2, Homeobox_disc2, Cp190_disc4, mod_mdg4__disc2, phol_known1, CTCF_disc2, CTCF_disc3, ttk_disc1, bab1_disc3)
(da_known1, twi_disc3, br_disc3, twi_known1, twi_known2, **twi_ef**, twi_disc2)
(sna_known3, sna_known4, sna_known8, twi_disc5, Homeobox_disc1) - discarded as too similar to the known twi motif
(h_disc3, run_disc1) - discarded as shifted versions of run_disc2 / Chro_disc3
(shn_disc1, kni_disc1, tll_disc1, sna_disc1, twi_disc1, Med_disc1, Homeobox_disc5, gt_disc1, D_disc2, da_disc1, Mad_disc1, **hb_disc1**, Kr_disc5, Med_disc2)
(brm_disc2, trx_disc3,run_disc2, trem_disc3, **Chro_disc3**, CBP_disc3, trem_disc2)
(EcR_disc2, EcR_disc4) - discarded as other motifs were used for EcR
(brm_disc1)
(Homeobox_disc8)
(**tin_ef**)
(kni_known5)
(BEAF-32_disc2)
(shn_disc2)
(h_disc2)

Selection of human TFs

The initial set of TF binding motifs was selected from the ENCODE motif repository [60] using the following criteria: correlation between median conservation (grep) scores and information content >= 0.5; slope of the regression line >= 0.12; number of ChIP peaks for this TF in at least one cell type >= 1300; number of TF ChIP peaks with motif(s) >= 120; number of non-overlapping motifs mapping to TF ChIP regions >= 150. This filtered for 322 out of the total 498 motifs for 68 out of 73 TFs. These motifs were then clustered based on Pearson correlation using STAMP [71, 72], resulting in clusters listed below (on cutting the tree at ~99% from root). From each of these clusters, a single best motif was selected based on the above parameters. If multiple motifs for a TF or multiple cell types passed this filter, a single best combination was chosen based on the same criteria, resulting in 36 TF datasets included in the final analysis. The included motifs are shown in bold in the clusters below and listed in Table S2.

(BRCA1_disc1 / CHD2_disc1 / **ZBTB33_disc1** / Ets_disc3 / ZBTB33_disc2)
(Mxi1_disc1 / RFX5_known2 / SREBP_disc1 / **RFX5_disc1** / RFX5_known3 / RFX5_known4 / Myc_disc4)
(RXRA_known3 / **RXRA_known4**)
(**ZEB1_known3** / ZEB1_known5 / TCF12_disc1)
(Irf_known6 / **Irf_known9** / Irf_known5 / Irf_disc3 / Irf_known1 / Irf_known2 / Irf_known3 / STAT_disc3 / PRDM_disc1)
(Irf_known7 / Irf_known10 / Irf_known11) – discarded as another motif was selected for Irf
(**Pou5f1_known1** / Pou5f1_known2 / Pou5f1_disc1 / Nanog_disc2)
(Maf_disc2 / Maf_known3 / **Maf_known4**)
(**Pbx3_disc2** / Pbx3_disc3 / SP2_disc2)
(CEBPB_known1 / CEBPB_known6 / p300_disc2 / CEBPB_known3 / CEBPB_known5 / **CEBPB_disc1** / CEBPB_known2)
CEBPB_known4 – discarded as another motif was used for CEBPB
(ELF1_known1 / Pax-5_disc4 / PU.1_disc1 / p300_disc10 / Ets_known1 / Ets_known9 / Ets_known2 / Ets_known3 / **Ets_known5** / BCL_disc1 / Egr-1_disc2 / Ets_known4 / Ets_disc2 / ELF1_disc1 / Ets_known8 /

Ets_known6 / Ets_known7 / STAT_known13)
(BCL_disc4 / Pax-5_disc3 / p300_disc5 / Mef2_disc2 / AP-1_disc8 / GATA_disc3) – discarded as too similar to Ets
(TATA_disc7 / GR_disc6) – discarded as another motif was used for GR; TATA not used in the analysis
(STAT_known6 / **STAT_known10** / STAT_known12 / STAT_known7 / STAT_disc1 / STAT_known14)
(Irf_disc4 / SP1_disc3 / SP1_known1 / SP1_known2 / SP1_known4) – discarded as poorly enriched at the peaks
(**TR4_disc1** / AP-1_disc6)
TCF12_disc6 – discarded as poorly enriched at the peaks
(**NFkB_disc1** / NFkB_known1 / NFkB_known2 / NFkB_known6 / NFkB_known5 / NFkB_known3 / NFkB_known4 / BCL_disc6)
(NFkB_disc3 / NFkB_disc4 / STAT_disc5) – discarded as other motifs were selected for both NFkB and STAT
AP-1_disc4 – discarded as another motif was selected for AP-1
(Pax5_known1 / **Pax5_disc2**)
(SIX5_disc1 / Znf143_disc2 / SIX5_disc2 / **Znf143_disc1** / Ets_disc1 / Znf143_disc3 / SETDB1_disc1 / Ets_disc7)
(GR_known1 / GR_disc1 / GR_known5 / **GR_known7** / GR_known8 / GR_known10 / GR_known3 / GR_known4 / GR_known9)
(**BATF_disc1** / AP-1_known1 / BAF155_disc1 / Maf_known1 / NF-E2_known1 / NF-E2_disc1 / Maf_disc1 / AP-1_known3 / Ap-1_known4 / GATA_disc2 / Myc_disc3 / RXRA_disc3 / AP-1_disc3 / PRDMI_disc2 / TCF4_disc1 / GR_disc2 / BATF_disc1 / BCL_disc2 / STAT_disc2 / p300_disc1 / KAP1_disc1 / AP-2_disc1 / HMGN3_disc1 / Irf_disc2 / Mef2_disc3)
AP-1_disc5 – discarded as another motif selected for AP-1
(RXRA_known3 / RXRA_known4 / ERalpha-a_known1 / ERalpha-a_disc2 / **ERalpha-a_disc1** / ERalpha-a_disc3)
(**AP-1_disc1** / ATF3_known1 / ATF3_known9 / ATF3_known7 / ATF3_known8 / E2F_disc1 / TATA_disc2 / HEY1_disc1)
(ERalpha-a_disc4 /Sin3Ak-20_disc6) – discarded due to low complexity
(RXRA_known1 / HNF4_known1 / **HNF4_known3** / TR4_disc2 / HNF4_known4 / RXRA_known6 / RXRA_disc1 / HNF_known8 / HNF4_disc1)
(**TCF4_known1** / TCF4_known3 / TCF4_disc2)
SP1_disc2 – discarded due to poor enrichment
(HNF4_known2 / HNF4_disc2 / HNF4_disc3) – discarded as another motif was used for HNF4
(TAL1_known1 / TAL1_known2 / GATA_disc4) – discarded due to poor enrichment at TAL1 bound regions; another motif was used for GATA
(Myc_known1 / Myc_known7 / **Myc_disc2** / Mxi1_disc2 / Myc_known4 / Myc_known9 / Myc_known8)
(Mxi1_known1 / BHLHE40_known2 / BHLHE40_disc1 / Myc_disc1 / ATF3_disc1 / NF-E2_disc2 / Myc_known5 / Myc_known6 / Sin3Ak-20_disc2 / BHLHE40_known1 / ATF3_disc2) – discarded as too similar to the selected Myc_disc2 motif
(Rad21_disc2 / Rad21 _disc4) – discarded as these motifs are a part of CTCF motif
(**CTCF_known1** / CTCF_disc1 / Rad21_disc1 / SMC3_disc1 / CTCF_disc5 / RXRA_disc2 / Rad21_disc5 / CTCFL_disc1 / ZBTB7A_disc1)
(**YY1_known3** / YY1_disc1 / THAP1_disc1 / TATA_disc1 / YY1_disc2 / YY1_known4 / YY1_known5)
(**NRSF_known1** / NRSF_known3 / NRSF_disc1 / Sin3Ak-20_disc1 / HDAC2_disc3 / NRSF_disc2 / BCL_disc3 / Sin3Ak-20_disc3)
(NRSF_disc3 / Sin3Ak-20_disc7 / Ets_disc5 / Sin3Ak-20_disc4) – discarded as these motifs are part of NRSF motif
(Znf143_known1 / Egf-1_disc1 / STAT_known11) – discarded due to similarity to the selected STAT_known10
Nanog_disc3 – discarded due to poor enrichment
(EBF_known1 / **EBF_known2** / EBF_disc1)
AP-2_known4
(Egr-1_known1 / **Egr-1_known4** / Egr1_known2 / Egr-1_known5)
(SP1_known1 / SP1_disc3 / SP1_known1 / Irf_disc4 / TATA_disc4 / SP1_known4) – discarded due to low complexity
(Myc_disc9 / YY1_disc3 / E2F_disc7 / TATA_disc10 / ELF1_disc2 / BCL_disc10 / Ets_disc9 / BCL_disc8 / YY1_disc4) – discarded due to low complexity
(Nrf1_known1 / **Nrf1_disc1** / E2f_disc2 / SETDB1_disc2 / Egr-1_disc3)
(**E2F_known1** / E2F_known2 / E2F_known5 / E2F_disc3)
(**SRF_known1** / SRF_known5 / SRF_known2 / SRF_known3 / SRF_known4 / SRF_disc1 / SRF_known6)
(Mef2_known1 / Mef2_known6 / Mef2_known3 / **Mef2_known2** / Mef2_known5 / Mef2_disc1 / TATA_known3 / TATA_known4 / TATA_known5)
(GATA_known1 / GATA_known4 / HDAC2_disc1 / HMGN3_disc2 / GATA_disc1 / GATA_known13 / GATA_known10 / GATA_known2 / GATA_known9 / GATA_known12 / **GATA_known14** / TAL1_disc1 / CCNT2_disc1)
(GATA_known3 / GATA_known6) – discarded as another motif was used for GATA
GATA_known11 – discarded as another motif was used for GATA
(**Pou2f2_known1** / Pou2f2_known6 / Nanog_disc1 / Pou2f2_known7 / Pou2f2_disc1 / Pou2f2_known10 / Pou2f2_known8 / TATA_disc9 / Pou2f2_known2 / E2F_disc6)
(Pou2f2_known5 / Foxa_disc2) – discarded as other motifs were used for Pou2f2 and Foxa
(Foxa_known1 / Foxa_known4 / **Foxa_known3** / Foxa_disc1 / p300_disc3 / HDAC2_disc2 / Foxa_known2 / HNF4_disc4)
(YY1_known2 / YY1_known6 / BATF_disc3) – discarded as other motifs were used for YY1 and BATF
(NF-Y_known1 / **NF-Y_disc1** / SP1_disc1 / RFX5_disc2 / Pbx3_disc1 / Ap-1_disc2 / Irf_disc1 / E2F_dosc4 / NF-Y_known3 / NF-Y_known2 / TATA_disc6 / SP2_disc1)
